# Sound Spectrum Influences Auditory Distance Perception of Sound Sources Located in a Room Environment

**DOI:** 10.3389/fpsyg.2017.00969

**Published:** 2017-06-22

**Authors:** Ignacio Spiousas, Pablo E. Etchemendy, Manuel C. Eguia, Esteban R. Calcagno, Ezequiel Abregú, Ramiro O. Vergara

**Affiliations:** ^1^Laboratorio de Dinámica Sensomotora, Departamento de Ciencia y Tecnología, CONICET, Universidad Nacional de QuilmesBernal, Argentina; ^2^Laboratorio de Acústica y Percepción Sonora, Escuela Universitaria de Artes, CONICET, Universidad Nacional de QuilmesBernal, Argentina

**Keywords:** psychoacoustics, auditory perception, distance perception, spectrum, reverberation

## Abstract

Previous studies on the effect of spectral content on auditory distance perception (ADP) focused on the physically measurable cues occurring either in the near field (low-pass filtering due to head diffraction) or when the sound travels distances >15 m (high-frequency energy losses due to air absorption). Here, we study how the spectrum of a sound arriving from a source located in a reverberant room at intermediate distances (1–6 m) influences the perception of the distance to the source. First, we conducted an ADP experiment using pure tones (the simplest possible spectrum) of frequencies 0.5, 1, 2, and 4 kHz. Then, we performed a second ADP experiment with stimuli consisting of continuous broadband and bandpass-filtered (with center frequencies of 0.5, 1.5, and 4 kHz and bandwidths of 1/12, 1/3, and 1.5 octave) pink-noise clips. Our results showed an effect of the stimulus frequency on the perceived distance both for pure tones and filtered noise bands: ADP was less accurate for stimuli containing energy only in the low-frequency range. Analysis of the frequency response of the room showed that the low accuracy observed for low-frequency stimuli can be explained by the presence of sparse modal resonances in the low-frequency region of the spectrum, which induced a non-monotonic relationship between binaural intensity and source distance. The results obtained in the second experiment suggest that ADP can also be affected by stimulus bandwidth but in a less straightforward way (i.e., depending on the center frequency, increasing stimulus bandwidth could have different effects). Finally, the analysis of the acoustical cues suggests that listeners judged source distance using mainly changes in the overall intensity of the auditory stimulus with distance rather than the direct-to-reverberant energy ratio, even for low-frequency noise bands (which typically induce high amount of reverberation). The results obtained in this study show that, depending on the spectrum of the auditory stimulus, reverberation can degrade ADP rather than improve it.

## Introduction

Perceiving accurately the location of a sound source is an essential capability of the human hearing system, enhanced through selective pressure due to its survival value (when the source is out of view or occluded, the auditory modality often plays a crucial role on assessing the location of the source). In addition to the perceived source direction, human hearing is sensitive to the source distance.

Our everyday experience shows us that there are large variations in the stimulus intensity and quality depending on the distance to an acoustic source that are potential cues for distance estimation. Among those, sound intensity is a primary cue based on the variation of this magnitude following the inverse-squared distance law in the free field (Coleman, 1963). In reverberant environments, there is also a systematic relation between the distance to the source and the reverberation amount relative to the level of the direct sound (energy that is transmitted directly from the source to the listener without interacting with any surfaces of the environment; Mershon and Bowers, 1979), leading to another relevant distance cue: the direct-to-reverberant energy ratio (DRR). In addition, cumulated evidence shows that, for near-field sources located outside the median plane, auditory distance perception (ADP) relies on low-frequency interaural level differences, an acoustical cue that rapidly increases its relative importance when the source approaches within 1 m of the listener's head (Brungart, 1999; Brungart and Rabinowitz, 1999; Brungart et al., 1999; Kopčo and Shinn-Cunningham, 2011). Finally, several studies have shown that spectral cues have a relevant influence on ADP both in the near field (Levy and Butler, 1978; Brungart, 1999; Brungart et al., 1999; Kopčo and Shinn-Cunningham, 2011) and in the far field (Coleman, 1968; Lounsbury and Butler, 1979; Butler et al., 1980; Petersen, 1990; Little et al., 1992). Both near- and far-field spectral cues are described in detail in the following section.

### Spectral cues

Spectral content provides a physically measurable cue for ADP only for very short (<1 m) or long (>15 m) distances to the source. In the first case, the diffraction of sound around the head causes a relative low-to-high frequency gain as the source approaches the listener (Brungart and Rabinowitz, 1999), providing a reliable cue to perceive distance in the near field. For long distances, as a sound wave propagates through the atmosphere, high-frequency components become more attenuated than low-frequency ones due to heat conduction, shear viscosity, and relaxation losses (Bass et al., 1995), low-pass filtering sound coming from distant sources. However, this effect is moderate (<0.2 dB/m for 8 kHz in normal conditions of pressure and temperature), therefore the sound must travel distances >15 m for a listener to detect changes in the sound spectrum (Ingard, 1953; Blauert, 1997). No measurable ADP cues were studied for sounds located in the range 1–15 m (Kolarik et al., 2015) since low-frequency head-diffraction-induced changes are too small to be detected for distances over 1 m and the sound has not traveled far enough for the high-energy loss to be detected for distances <15 m.

Several studies have examined the effect of the spectral cues on ADP. In the near field, Brungart (1999) showed that, in anechoic environments, ADP accuracy is comparable between broadband and low-pass filtered stimulus (<3 kHz), while, for a high-pass filtered stimulus (>3 kHz) the accuracy was significantly reduced, showing that accurate distance judgments for proximal sound sources required components below 3 kHz, a result that is consistent with the low-pass filtering caused by head diffraction. In a more recent work, Kopčo and Shinn-Cunningham (2011) employed stimuli covering different regions of the audible spectrum to study the effect of spectral cues on ADP of frontal and lateral near-field sources in a virtual reverberant environment. Their results showed that, like what was reported by Brungart (1999) in the free field, the spectral characteristic that most strongly influenced near-field ADP was the lowest frequency present in the stimuli.

In the far field, several studies have shown that stimuli are judged to be more distant as their high-frequency content decreases relative to the low-frequency content (Coleman, 1968; Lounsbury and Butler, 1979; Butler et al., 1980; Petersen, 1990). Interestingly, this effect has been also observed for intermediate distances (<15 m) where the changes in spectral content could not be produced by air absorption. Butler et al. (1980) examined this issue by comparing estimates of distances for broadband, high-pass (cutoff frequencies of 6.0, 4.0, and 2.0 kHz) and low-pass (cutoff frequencies of 2.0, 1.0, or 0.5 kHz) noise bands recorded using a fixed loudspeaker, both in an anechoic and an echoic room, and presented to the participants through headphones. High-pass stimuli were systematically judged closer than broadband stimuli, and broadband stimuli were systematically judged closer than low-pass stimuli in both environments, and the effect was found stronger in the echoic room. The authors discussed a possible explanation for this last difference by considering spectral changes due to acoustic reflections, but without further advancing in this hypothesis. Little et al. (1992) showed, by using stimuli like that produced by variations in physical distance (broadband noises low-pass filtered at 5, 6, and 6.7 kHz), that a reduction on the high-frequency content is associated to a larger reported distance only when the individuals can compare the stimuli with one another, but not for a first presentation of the stimulus. The authors concluded that ecologically appropriate variations in spectral content can act as a relative cue (but not as an absolute cue) for perceived auditory distance.

In reverberant environments, spectral content is affected by the frequency response of the room, a fact that has long been studied in the context of sound reproduction systems (Fazenda et al., 2015), but that has not been addressed before in ADP. The frequency response of a room at high frequencies is mainly determined by the absorption properties of the surrounding surfaces, and at low frequencies is dominated by the modal resonances (standing waves) that become sparse, narrower and with longer decays as the frequency is lowered (Kuttruff, 2016). In fact, the modal resonances of the room could produce strong frequency-dependent variations in sound intensity at the listener's ears (Antsalo et al., 2004; Fazenda et al., 2015), thus potentially affecting the apparent distance of the source through the intensity cue.

The spectrum of auditory stimulus also affects the precision to perceive changes in DRR (Larsen et al., 2008). Previous studies showed that listeners are better at discriminating changes in DRR for stimuli containing low frequencies than for stimuli with only high-frequency content; in addition, reductions in frequency bandwidth lead to statistically significant increases in just noticeable differences of DRR (Zahorik, 2002b; Larsen et al., 2008). Based on these evidences, we consider it likely that changes in the spectral content of the stimulus could affect the perceived distance of the sound source in a reverberant environment even at intermediate distances (between 1 and 15 m), even if not directly, through the intensity or DRR cues.

### The aim of this study

Previous studies on the effect of spectral content on ADP focused on the physically measurable cues occurring either in the near field (due to head diffraction) or when the sound travels distances >15 m (due to air absorption). How the interaction between the spectrum of the sound and the reverberation of the room affects ADP was only addressed for near-field sources by Kopčo and Shinn-Cunningham (2011), while, for intermediate distances, this relation was explored indirectly (Butler et al., 1980). However, in everyday circumstances, humans often need to estimate the distance to sound sources located in a room, under the influence of reverberation and at moderate distances. The question of how the spectral content of the stimulus emanating from a sound source inside a room can affect its perceived distance is not yet fully answered. A complementary question that arises is whether the frequency response of the room can also influence the perceived distance or not, an issue that was not addressed before.

To answer these two questions, we first conducted two ADP experiments. In Experiment 1 we used stimuli with the simplest spectral content (pure tones with frequencies 0.5, 1, 2, and 4 kHz); and in Experiment 2, stimuli consisted of continuous broadband (control stimulus) and bandpass-filtered pink-noise clips with various central frequencies (0.5, 1.5, and 4 kHz) and bandwidths (1.5, 1/3, and 1/12 octaves). Secondly, and to assess the effect of modal resonances on the acoustical field, we measured the binaural intensity (BI) and the DRR for each distance and stimulus used in Experiment 2. Finally, we studied if the changes observed in the subjective distance judgments could be explained by changes in the measured acoustical magnitudes.

## Materials and methods

### Testing environment

All experiments were performed in a semi-reverberant room of size 12 × 7 × 3 m (length × width × height) with walls covered by sound-absorbing panels (pyramid polyurethane acoustical foam, 50 mm), the floor by a carpet, and the ceiling by fiberglass acoustic panels. The mean reverberation time of the room, obtained using an exponential sweep (Farina, 2000) measured at the position of the listener and with the source located at the farthest position in the experiment (6 m, see Section Experimental Set-Up and Auditory Stimuli), is close to 0.3 s. As we are also interested in the frequency response of the room we report the reverberation time for each octave band (see Table 1). The background noise of the room is 19 dBA (measured with a RION NL-32 sound level meter at the position of the listener).

**Table 1:** Reverberation times of the experimental room.

**0.125 kHz**	**0.250 kHz**	**0.5 kHz**	**1 kHz**	**2 kHz**	**4 kHz**	**8 kHz**	**All octaves**
662 ms	395 ms	275 ms	304 ms	213 ms	97 ms	92 ms	298 ms

*Reverberation times of the experimental room in octave bands (ms), obtained by extrapolating the decay curves between −5 and −25 dB*.

### General procedure

The procedure consisted of presenting auditory stimuli at one of the six distances (*D* = 1, 2, 3, 4, 5, and 6 m). Participants were instructed to judge the apparent egocentric distance to the auditory target (a loudspeaker) using a free scale of meters with no limits on range nor precision. The responses were reported verbally. Participants were not informed of the possible values of *D*.

Each kind of stimulus (described in Section Experimental Set-Up and Auditory Stimuli) was tested in an independent block. Thus, each experiment had as many blocks as types of stimuli tested (4 blocks in Experiment 1 and 10 blocks in Experiment 2). To wash-out learning-related biases, the order in which auditory stimuli were tested was fully randomized, except in Experiment 2 where the pink noise (control stimulus) was always presented first. Within each block, stimuli were presented three times for each distance in random order, giving a total of 18 trials per block. Only one verbal response was made per trial, and participants did not receive any feedback regarding his/her performance. Each block lasted ~6 min, and was followed by a pause of 5 min.

Instructions were given to participants inside the testing room and with lights on. This allowed them to perform a visual inspection of the environment and the experimental set-up; nevertheless, participants were not explicitly informed of their dimensions. Previous results obtained in our laboratory, and therefore in the same acoustical environment, showed that visual inspection of the room helps subjects to decrease their bias in the response (Calcagno et al., 2012). Once instructed, participants were seated in a chair positioned at the zero point, where they were blindfolded before starting the experiment.

### Experimental set-up and auditory stimuli

**Figure 1:**
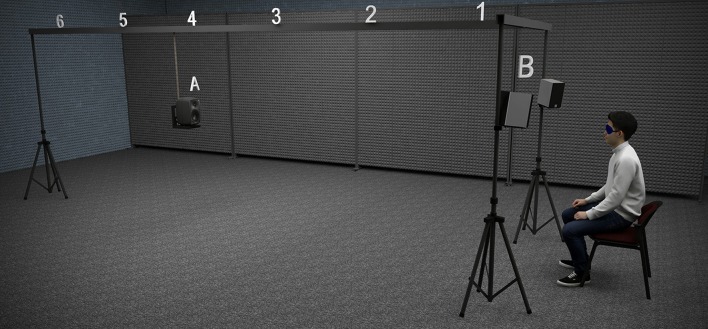
Illustration of the experimental set-up. The set-up consisted of a mobile test loudspeaker **(A)** suspended from a metal rail, and a masking system **(B)** formed by two fixed loudspeakers located at both sides of the participant's head. The source positions for Experiments 1 and 2 (*D* = 1, 2, 3, 4, 5, and 6 m) are indicated above the metal rail.

The experimental set-up was identical to the one used in a previous paper (Calcagno et al., 2012). Figure 1 shows an illustration of the set-up. It consisted of a test loudspeaker (A) located in front of the participant, 1.2 m above the floor (approximately the height of a seated participant's ears) and suspended from a 6-m-long metal rail. The loudspeaker (Genelec 8020B bi-amplified 50 W) was free to move along the rail, allowing for the presentation of auditory stimuli from any distance within the available range. The set-up was completed by a masking system, consisting of two fixed loudspeakers (Edifier R1000TCN 25 W) located at both sides of the participant and pointing to his/her ears (B). Both the test loudspeaker and the masking system were controlled by a stereo sound card (MOTU 896 mk3) connected to a personal computer. The computer also controlled the psychophysical procedure by establishing the stimuli order. The straight line between the participant and the target loudspeaker was parallel to two walls, but slightly offset from the central line of the room.

The operation of the set-up was as follows. The test loudspeaker was displaced manually by one experimenter to one of the six possible source positions pseudo-randomly established by the computer for each trial, displayed in the computer screen. The rail had small markings for each position, only visible for the experimenter, which allowed accurate location of the test loudspeaker. To mask the sound produced by the manipulation of the test loudspeaker along the rail, before each trial a masking sound (12-s long) was presented through the masking system. We took this precaution since the sound produced by the displacement of the loudspeaker could serve as an undesired cue for the source distance. Moreover, the time required to move the loudspeaker from one position to the next could also serve as a relative cue. The duration of the masker (12 s) was set to a value that allowed the displacement of the test loudspeaker between the extreme positions (1 and 6 m) at a gentle pace. Two seconds after the end of the masking sound, the auditory stimulus was presented through the test loudspeaker. At this point the subject expressed the perceived distance verbally. Finally, the response was introduced in the computer by a second experimenter seated in the room. The experimenter in charge of moving the loudspeaker was present in the room the whole time but he only moved it toward the target position during the masking noise. Once the loudspeaker was located at the target distance, the experimenter remained still (one footstep behind the loudspeaker) until the beginning of the masking sound of the following trial.

All stimuli were digitally generated with a personal computer installed with Matlab (The MathWorks, Inc., Natick, Massachusetts, United States) at a sampling frequency of 44.1 kHz, and had a duration of 500 ms with onset and offset ramped by a raised cosine of 50 ms. Stimuli of Experiment 2 were generated by filtering a pink noise with digital Butterworth bandpass filters (function fdesign.bandpass in Matlab), geometrically centered at 0.5, 1.5, and 4 kHz, with bandwidths of 1/12, 1/3, and 1.5 octaves and slopes of 80 dB/octave. The center frequencies and lower and higher limits of the bandpass for all the noise bands are listed in Table 2. The sound level of the stimulus and masking sound was 70 dBA measured with a RION NL-32 sound level meter at the participant's position and the source located at 1 m.

**Table 2:** Details of the spectral content of the stimuli used in Experiment 2.

**Center frequency (kHz)**	**Bandwidth (octaves)**	**F-pass (kHz)**
0.5	1/12	0.489/0.515
	1/3	0.445/0.561
	1.5	0.297/0.841
1.5	1/12	1.457/1.544
	1/3	1.336/1.684
	1.5	0.892/2.523
4	1/12	3.886/4.117
	1/3	3.564/4.490
	1.5	2.378/6.727
Pink noise (PN)		0.02/20

*Center frequencies and lower and higher limits of the bandpass filter for all the noise bands used as stimuli in experiment 2. F-pass stands for the frequency at the edge of the start of the pass band filter*.

### Participants

A total of 23 volunteers (15 men, *M*_age_ = 26.0 y.o., *SD*_age_ = 6.5 y.o.) participated in the experiments. Although explicit measurements of auditory sensitivity were not performed, all participants reported normal hearing. The experiments were undertaken with the understanding and written consent of each subject, following the Code of Ethics of the World Medical Association (Declaration of Helsinki) and were approved by the Ethics Committee of the Universidad Nacional de Quilmes.

### Data analysis

Individual distance curves were obtained by calculating means and standard deviations on the individual responses across trials for each source distance. Average distance curves were obtained by repeating the process on the individual means. Individual ADP curves were analyzed for each stimulus by means of several statistics:

1. The Pearson product-moment correlation coefficients between physical and perceived source distances (as was done by Brungart, 1999; Brungart et al., 1999; Kopčo and Shinn-Cunningham, 2011);
2. the slopes of least-squares linear fits between source and response distances (Anderson and Zahorik, 2014); and
3. the average of the standard deviations (obtained from the average of the corresponding variances) across the sound source distances (as done in Kopčo and Shinn-Cunningham, 2011).

The three statistics were computed on a log-distance scale. The use of individual measures allowed us to use within-subjects models for the analysis of statistical trends.

Finally, across-subject measures were obtained by calculating the mean and standard deviation of the aforementioned statistics over subjects for each stimuli type. In the case of the correlation coefficients, the Fisher *z*-transformation:

$$\begin{array}{l} z=\tanh^{-1}\left(r\right) \end{array}$$

of the coefficient was calculated before the averaging process, to improve normality (Cohen et al., 2003).

All data were statistically analyzed at a significance level of 5%, and the correction of Holm–Bonferroni (family-wise type I error α_*fw*_ = 0.05; Holm, 1979) was used when multiple comparisons were performed. In all the within-subjects ANOVA's performed throughout this work, Mauchly's test was employed to evaluate whether the assumption of sphericity was met. When data failed to meet the assumption, the Greenhouse-Geiser correction was applied to the degrees of freedom of the *F*-statistic. In order to obtain a confidence interval for the effect size ($\eta_{p}^{2}$) that is equivalent to the ANOVA *F*-test of the effect, we employed a confidence coefficient of (1–2α), which in our case corresponds to a 90% confidence interval (Steiger, 2004).

### Acoustical measurements techniques

The recordings were made using the same mobile sound source as used for the experiments (Genelec 8020B) and a custom-designed binaural dummy head placed at the subject's location equipped with SP-TFB-2 intra-aural microphones (same as used in Spiousas et al., 2015).

The sound samples used for the recordings consisted of the same broadband and filtered pink-noise clips (500-ms long) used as stimuli in Experiment 2. In addition, we recorded an exponential sweep (0.02–20 kHz and 20-s long) to obtain the binaural room impulse response (BRIR) for each sound source position. Both the sound samples and the impulse responses were A-filtered since the recordings were made at the entrance of the dummy head's ear canal.

**Figure 2:**
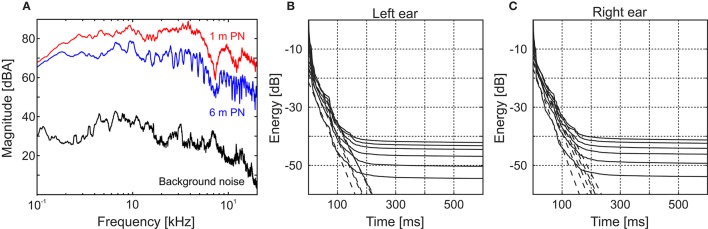
Testing environment acoustical analysis. (A) Magnitude spectra of the 500-ms pink noise clips at 1 and 6 m (red and blue lines, respectively) and the background noise (black line). The background noise spectrum was obtained using a 6.5-s-long recording at the listener position. **(B,C)** Cumulative energy decay functions for the binaural impulse responses corresponding to all the measured distances (1–6 m from bottom to top, respectively) obtained for the left **(B)** and right **(C)** ears. For both ears the flattening of the decay curve occurs around 150 ms, after the impulse response intensity has already decayed, at least, 40 dB.

Signal-to-noise ratio was calculated for all recorded stimuli obtaining global values always above 31.4 dB (mean = 37.6 dB, *SD* = 4.2 dB, range = 31.4–47.7 dB). As an example of the spectral profile of this relation, in Figure 2A we show the magnitude spectra of the recorded PN stimuli both in the closest (red) and farthest (blue) positions along with the background noise (black) present in the room. It is worth noting that, as the rest of the stimuli are filtered versions of this pink noise, the spectral shape of the signal-to-noise ratio remains the same.

### Acoustical magnitudes calculation

From the binaural recordings and the BRIRs of the dummy-head we derived two acoustical measures corresponding to the two most relevant cues in our experiment: sound intensity and direct-to-reverberant ratio. Other binaural and dynamical cues were not considered due to the static nature of the experimental setup and the range of distances involved (the source is located in front of the subject and in the far field but not too far for effects of the sound-absorbing properties of the air to be noticeable).

#### Binaural intensity

We calculated the BI by linearly adding the intensity of the signal reaching both ears as follows:

$$\begin{array}{lll} I_{l,r} & = & \frac{1}{L}\overset{L}{\underset{0}{\int }}p_{l,r}^{2}\left(t\right)dt\\ BI & = & 10log_{10}\left[{\left(I_{l}I_{r}\right)}^{1/2}/I_{ref}\right] \end{array}$$

where *I*_*l,r*_ stands respectively for the left and right ear individual intensity, *p*_*l,r*_*(t)* for the pressure field on each ear, *L* for the length of the recorded signal, and *I*_*ref*_ = 10^−12^ W/m^2^ is the reference intensity.

#### Direct-to-reverberant energy ratio

We defined the direct field by simply time-windowing the BRIR. Thus, we assumed the direct sound being comprised within the time interval of 1.2 ms after the arrival of the first wavefront, indicated by the first prominent peak in the BRIR. The remaining portion of the BRIR, corresponding to times >1.2 ms after the first peak, was considered as reverberant field. This time was chosen such that it separates the direct sound from all reflections in the BRIR (including the floor reflection) for all source distances.

Once the direct and reverberant fields were separated, we calculated the DRRs by convoluting each portion of the BRIR with the filtered noise bands, and then computing the ratio between the total energy contained in the two portions. Finally, we obtained a unique value of the DRR for each position and type of stimulus by averaging the ratios obtained for each ear.

One of the main concerns when dealing with DRR calculation in a noisy environment is not to consider the background noise as reverberant field. In order to assess if it was our case we calculated the cumulative energy decay functions (Schroeder, 1979) for pink noise (Figures 2B,C for the left and right ear, respectively). In these curves, even for the worst case (farthest source distance), the energy decays almost 40 dB before flattening. As a secondary check, we recalculated all the DRR values cutting the IRs at a time in which all the curves are still decaying (150 ms) and found that the differences were almost negligible (mean = 4.4 × 10^−3^ dB, *SD* = 5.0 × 10^−3^ dB, range = 0–2.1 × 10^−2^ dB).

## Results

### Psychophysical experiments

#### Experiment 1: apparent source distance for pure tones

**Figure 3:**
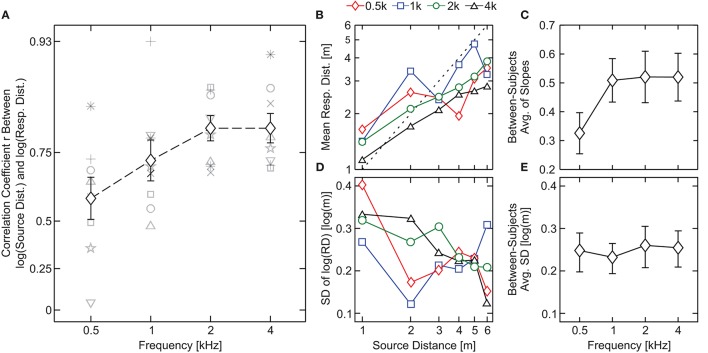
Results of Experiment 1. **(A)** Pearson linear correlation coefficient (*r*) between source distance and response distance (in log-scale) as a function of the stimulus type. Black symbols represent across-subject averages, shaded symbols show the individual subject data and bars denote standard errors. **(B)** Across-subject mean of the log-scale subjective distance judgments as a function of the physical distance to the source. Perfect performance is indicated by a black dashed line. **(C)** Across-subject average of the slopes obtained by means of least-squares linear fits between the source and the individual response distances in logarithmic scale. **(D)** Across-subject average of the responses' standard deviation as a function of the physical distance to the source. **(E)** Standard deviations collapsed over distances averaged across subjects. In **(C,E)** bars denote SEM.

Eight subjects participated in the experiment (7 men, *M*_age_ = 27.0 y.o., *SD*_age_ = 8.3 y.o.). The across-subject mean of the logarithm of subjective distance judgments in response to pure tones of 0.5, 1, 2, and 4 kHz are shown in Figure 3B as a function of the physical distance to the source. For 0.5- and 1-kHz tones there is a slight increase of the response with the distance, however, it is not clear that a monotonic relation exists between the physical and perceived distance. For example, for a pure tone of 0.5 kHz subjects did not differentiate the distance to sources located at 1 or 4 m (responses were 1.95 ± 0.61 and 2.16 ± 0.64 m, respectively). The same occurs for 1-kHz tones located at 2, 4, and 6 m (3.74 ± 1.29, 3.81 ± 0.63, and 3.58 ± 1.05 m, respectively). In contrast, as we increase the stimulus frequency (2- and 4-kHz tones) the results show a monotonically-increasing relation between the physical and perceived distance. Moreover, for stimuli of 2 and 4 kHz subjects tend to underestimate, in average, the distance to the source for *D* > 2 m and *D* > 1 m, respectively.

In order to quantify the performance of the subjects for each frequency, we calculated the Pearson linear Correlation Coefficient (*r*) between individual responses and target distances in logarithmic scale (see Brungart, 1999; Brungart et al., 1999; Kopčo and Shinn-Cunningham, 2011), which are displayed in Figure 3A. The results show that the correlation coefficient increases with stimulus frequency. A within-subjects ANOVA with “frequency” as fixed factor revealed an effect of the stimulus frequency on the correlation coefficients [*F*_(3, 21)_ = 3.8, *p* = 0.026 and $\eta_{p}^{2}$ = 0.35 with 90% CIs (0.029; 0.50)] along with a fairly large effect size, indicating that nearly 35% of the total variance observed in *r* is due to changes in the frequency.

The correlation coefficient depends on the magnitude of the change of the response with the distance, the variability of the response, or a variation of both (Brungart et al., 1999; Kopčo and Shinn-Cunningham, 2011). To examine the cause (or causes) of the effect of the frequency of the pure tones on the correlation coefficients, we analyzed the values of these variables for each kind of stimulus.

To quantify the magnitude of the change we obtained the slopes corresponding to least-squares linear fits between the source and the individual response distances in logarithmic scale. In Figure 3C we show the between-subjects average of the slopes (bars denote SEM). The lowest slope value was obtained in response to the 0.5-kHz pure tone (0.33 ± 0.07 m). For the remaining stimuli, the slopes were fairly similar (0.50 ± 0.08, 0.52 ± 0.09, and 0.52 ± 0.08 m for 1, 2, and 4 kHz, respectively). A within-subjects ANOVA with factor “frequency” was performed on the slopes resulting in no significant effect [*F*_(3, 21)_ = 2.2; *p* = 0.12].

In Figure 3D, averages of the responses' standard deviations for each subject are shown as a function of the distance to the source. If we collapse the variability across distances (i.e., if we average the standard deviation of the response over all distances), we obtain a single measure of the variability for each stimulus. Collapsed variability is shown in Figure 3E. A within-subjects ANOVA with factor “frequency” resulted in no significant effect of this factor [*F*_(3, 21)_ = 0.169; *p* = 0.92] on the collapsed variability.

#### Experiment 2: apparent source distance for noise-bands

The aim of this experiment was to study whether the center frequency and bandwidth of auditory stimuli affects ADP. Details of the stimuli spectral content are shown in Table 2. Fifteen subjects participated in the experiment (8 men, *M*_age_ = 25.5 y.o., *SD*_age_ = 5.6 y.o.), none of which participated in Experiment 1.

**Figure 4:**
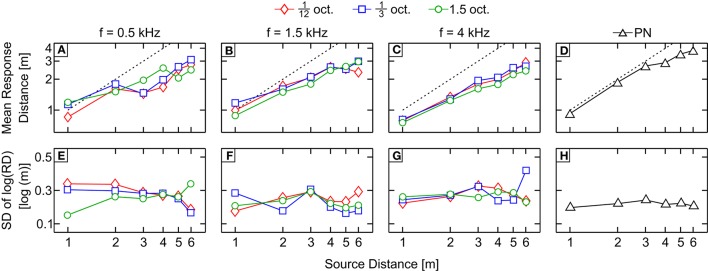
Results of Experiment 2 (average distance and variability). **(A–D)** Across-subject mean of the logarithm of the subjective distance judgments as a function of the physical distance to the source in response to bands centered at 0.5, 1.5, and 4 kHz (**A–C**, respectively) and pink noise **(D)**. Perfect performance is indicated by a black dashed line. **(E–H)** Averages of the responses standard deviations obtained in response to bands centered at 0.5, 1.5, and 4 kHz (**E–G**, respectively) and pink noise **(H)**. The code symbols of different bandwidths are red diamonds, blue squares, and green circles for 1/12, 1/3, and 1.5 octave, respectively.

Figures 4A–D show the across-subject mean subjective distance judgments in response to filtered pink noise bands centered at 0.5, 1.5, and 4 kHz (Figures 4A–C, respectively) with bandwidths 1/12, 1/3, and 1.5 octave; and to pink noise (Figure 4D), as a function of the physical distance to the source (both in log scale).

The response obtained with PN was accurate for the first three distances while the distance to the source was underestimated for distances *D* = 4, 5, and 6 m. The responses for the remaining conditions were less accurate and showed a common pattern: distance was underestimated for sources farther than 1 m except for bands centered at 4 kHz, for which the responses show underestimation at all tested distances. Stimuli centered at 0.5 kHz showed a slight increase of the response with the distance and, unlike the bands centered at 1.5 and 4 kHz, they did not show a monotonic increase with increasing distance from the sound source. Responses for bands centered at 0.5 kHz with bandwidths 1/12 and 1/3 oct. show a non-homogeneous increase, with jumps and even decreases in the perceived distance when the distance from the source also increases. For example, the reported distance for 1/3-oct. bands was 2.11 ± 0.61 and 1.65 ± 0.47 m for source distances of 2 and 3 m, respectively.

**Figure 5:**
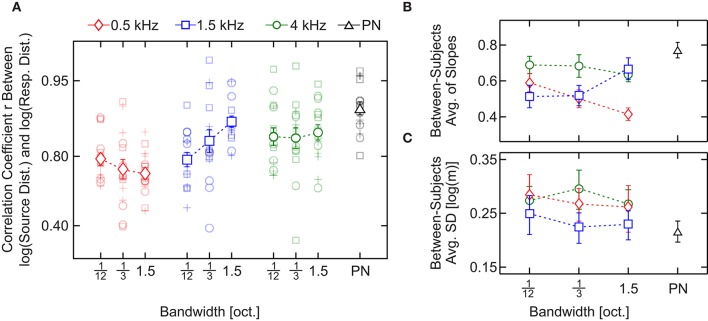
Results of Experiment 2 (statistical analysis). **(A)** Pearson linear correlation coefficient (*r*) between source distance and response distance (in log scale) as a function of the stimulus type. Across-subject averages in response to noise bands centered at 0.5, 1.5, and 4 kHz and pink noise are indicated with red diamonds, blue squares, green circles, and a black triangle, respectively. Shaded symbols show the individual *r*'s. **(B)** Across-subject average of the slopes obtained by means of least-squares linear fits between the source and the individual response distances in logarithmic scale. **(C)** Across-subject standard deviations collapsed over distances. In **(B,C)** symbols correspond to the same conditions described in **(A)**. In all panels bars denote SEM.

Performance, measured as the Pearson linear correlation coefficient (*r*) between individual responses and source distances in log scale for each stimulus, is shown in Figure 5A. The results of the analysis show a consistent effect, as observed in Experiment 1: *r* values are lower for bands centered at 0.5 kHz [mean across bandwidth = 0.759, 95% CIs (0.754, 0.763)] than for bands centered at 4 kHz [mean across bandwidth = 0.861, 95% CIs (0.857, 0.865)]. The greatest value of r was obtained in response to PN [mean = 0.916, 95% CIs (0.894, 0.934)]. Interestingly, the values of r obtained in response to bands centered at 0.5 kHz (*r* seems to decrease with the bandwidth) and 1.5 kHz (*r* seems to increase with the bandwidth) suggest an opposite effect of bandwidth on performance.

A two-way, repeated-measures ANOVA with within-subjects factors “center frequency” and “bandwidth” was performed on the correlation coefficients. The test yielded a significant main effect of the center frequency [*F*_(2, 28)_ = 16, *p* = 2.2 × 10^−5^, $\eta_{p}^{2}$ = 0.54, 90% CIs (0.28, 0.65)] but not of the bandwidth [*F*_(2, 28)_ = 1.1, *p* = 0.33] nor the interaction [*F*_(4, 56)_ = 2.4, *p* = 0.057]. Since there is a statistical tendency in the interaction, we performed a linear regression analysis to characterize the influence of the bandwidth on the correlation coefficient for each center frequency. For the 1.5-kHz bands we obtained a positive (and significantly non-zero) slope [mean slope = 0.229, SEM = 0.0638, *p*-value = 0.0030] but for the 0.5- and 4-kHz bands the slope was not significantly different from zero [500 Hz: mean slope = −0.0797, SEM = 0.0515, *p*-value = 0.14; 4 kHz: mean slope = 0.0340, SEM = 0.0689, *p*-value = 0.63]. These results indicate that, while for 0.5- and 4-kHz bands there is no observable effect of the bandwidth, for 1.5-kHz bands an increase in bandwidth entails an increase in the correlation coefficient.

Between-subjects average of the slopes are shown in Figure 5B. Like for the *r* values, the response for bands centered at 0.5 and 4 kHz presented the lowest and the highest slope values (with the exception of PN), respectively, and the slopes in response to bands centered at 0.5 kHz seem to decrease with the bandwidth while they seem to increase for bands centered 1.5 kHz. Finally, the slope obtained in response to bands centered at 4 kHz does not seem to depend on the bandwidth of the auditory stimulus.

A two-way, repeated-measures ANOVA with within-subjects factors “center frequency” and “bandwidth” was performed on the slopes. Similarly to that observed for r values, we found an effect of the center frequency [*F*_(2, 28)_ = 10, *p* = 4.2 × 10^−4^, $\eta_{p}^{2}$ = 0.43, 90% CIs (0.16, 0.57)] but not of the bandwidth [*F*_(2, 28)_ = 0.69, *p* = 0.51]. However, in this case we found a significant effect of the interaction between the factors [*F*_(4, 56)_ = 5.4, *p* = 9.8 × 10^−4^, $\eta_{p}^{2}$ = 0.28, 90% CIs (0.043, 0.44)]. The simple-effect analysis (Holm–Bonferroni corrected; Holm, 1979) showed a significant effect of the frequency for bands with 1.5 oct. bandwidth [*F*_(2, 28)_ = 16, *p* = 1.8 × 10^−5^, $\eta_{p}^{2}$ = 0.54, 90% CIs (0.29, 0.66)]; and of the bandwidth for bands centered at 0.5 kHz, [*F*_(2, 28)_ = 7.43, *p* = 2.6 × 10^−3^, $\eta_{p}^{2}$ = 0.35, 90% CIs (0.092, 0.50)].

In Figures 4E–H averages of standard deviations of the response of each subject are shown. We collapsed the variability between distances to obtain a single measure of the variability for each stimulus (Figure 5C). Stimuli centered at 1.5 kHz showed the lowest values of SD, while stimuli centered at 4 kHz showed the highest. The SD obtained with stimuli centered at 0.5 kHz showed intermediate values.

A two-way, repeated-measures ANOVA with within-subjects factors “center frequency” and “bandwidth” was performed on the collapsed SD, which revealed a significant effect of the center frequency [*F*_(1.35, 18.9)_ = 4.13, *p* = 0.046, $\eta_{p}^{2}$ = 0.23, 90% CIs (0.002, 0.434)] but not of the bandwidth [*F*_(2, 28)_ = 1.21, *p* = 0.31]. The analysis also showed a non-significant effect of the interaction between the factors [*F*_(4, 56)_ = 0.634, *p* = 0.64].

Results obtained in Experiment 2 show an effect of the frequency on the three obtained measures of the performance: correlation coefficient, slope and intra-subject standard deviation. Although both the slope and the standard deviation were significantly affected by the frequency of the stimuli, the fact that the frequency induces a similar trend in the correlation coefficients and the slope suggests that the observed effect of the frequency on the correlation could account mainly for the changes in the compression of the response. Regarding the effect of the bandwidth on the responses, the results were ambiguous. First, analysis of variance performed on the correlation coefficients showed a non-significant main effect of bandwidth. However, the statistical tendency in the interaction observed in the correlation coefficient in Experiment 2, the positive slope in the linear regression analysis for 1.5-kHz bands, and the significant effect of bandwidth on the slope for 0.5-kHz bands indicate that further analysis is needed to better understand the influence of bandwidth on the subjects' responses.

#### Comparisons with pink noise as a control

The results of Experiment 2 showed that the most accurate response was obtained for PN, showing a greater correlation and slope and a lower standard deviation across subjects. In this section, we compare the responses obtained in Experiment 2 for PN (control stimulus) with the response obtained for the 9 filtered noise bands. We performed two-tailed paired *t*-tests on the correlation coefficients using Dunnett's test for controlling the family-wise type I error (Dunnett, 1964), which is an appropriate procedure for comparing several treatments against a control. Results are displayed in Table 3. All except the 1.5-kHz, 1.5-oct. band were significantly different from the control, which is consistent with a non-additive effect of frequency and bandwidth on the correlation.

**Table 3:** Comparisons with pink noise as a control.

**Center frequency (kHz)**	**Bandwidth (octaves)**	***t*-value**	***p*-value**
0.5	1/12	6.42	2.42 × 10^−8^^*^
	1/3	7.73	6.42 × 10^−12^^*^
	1.5	8.27	2.68 × 10^−13^^*^
1.5	1/12	6.53	5.73 × 10^−9^^*^
	1/3	4.13	5.16 × 10^−4^^*^
	1.5	1.69	0.437 (ns)
4	1/12	3.60	3.63 × 10^−3^^*^
	1/3	3.77	2.06 × 10^−3^^*^
	1.5	3.07	1.92 × 10^−2^^*^

*Comparisons between the correlation coefficients obtained for pink noise with those obtained for the filtered bands, by means of t-tests. For each comparison, the t- and p-value are reported following Dunnett's method for comparing several treatments against a control. Asterisks indicate statistically significant differences at the (family-wise) 0.05 level. The t-value corresponds to 9 comparisons and 126 degrees of freedom (15 participants)*.

### ADP acoustical cues

#### Binaural intensity

**Figure 6:**
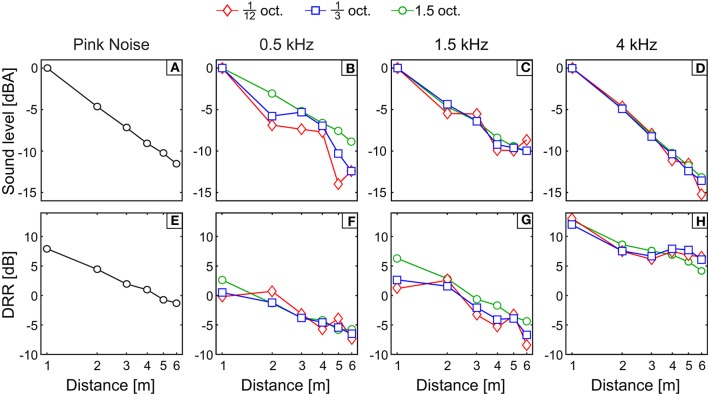
Acoustical magnitudes. **(A–D)** Binaural intensity and **(E–H)** direct-to-reverberant energy ratio (DRR) as a function of the physical distance to the sound source. In the rightmost panels **(D,H)** we can see these magnitudes for a pink noise stimulus. The rest of the panels **(A–C,E–G)** present the results for the pink noise filtered bands used as stimuli in Experiment 2 ordered by increasing center frequency. The code symbols of different bandwidths are red diamonds, blue squares, and green circles for 1/12, 1/3, and 1.5 octave, respectively. In panels **(A–D)** the curves were normalized to BI = 0 dB for the nearmost source.

The binaural intensity for PN and the filtered bands are plotted as a function of distance on Figure 6. Since sound intensity can be considered as a relative ADP cue (Mershon and Bowers, 1979), the global intensity for all bands was set as 0 dB at 1 m, letting us to focus on the relative decay instead of on the absolute values.

For the 1/12- and 1/3-oct. bands with center frequency of 0.5 kHz (Figure 6B), the BI shows a non-monotonous decay with abrupt jumps and even increases of intensity with distance. For example, for the 1/3-oct. band, when the source changes from 2 to 3 m, the BI increases 0.45 dB instead of decreasing. This behavior, that seems counterintuitive at first sight, is related to the existence of prominent and sparse modal resonances in the lower part of the frequency response of the room. As the frequency of the sound source is lowered, there are two effects general to all rooms, that cause the modal resonances to be more noticeable: On the one hand, the sound absorption at the walls, floor, and ceiling is reduced for lower frequencies, therefore the resonance peaks in this part of the spectrum become narrower in bandwidth and higher in amplitude and, on the other hand, the number of modal resonances per octave of the room decrease as the frequency is lowered (Kuttruff, 2016). Hence the frequency response of the room is not uniform for the lower frequency region. Moreover, for sounds with frequencies corresponding, or neighboring, to the modal-resonance frequency, where a standing wave is excited, the spatial distribution of the energy is also non-uniform. The standing wave creates spatial regions with peaks (antinodes) and dips (nodes) in the sound intensity. As a consequence, when the source emits low-frequency narrowband noise, the room response will be non-uniform both in frequency and space, since only a few modal resonances will be excited. In that situation, if the listener is seated close to a node or antinode of the created standing waves, the BI will be significantly lowered or increased, respectively, compared to the neighboring region.

For the 1/12-oct. bands, as the central frequency increases, the decay becomes more homogeneous because: (1) The intensity of the reverberant field does not account much on the global intensity due to the reduction of the reverberation for frequencies above 0.5 kHz; and (2) the resonances of the room become more dense and wide, hence the frequency response of the room becomes more homogeneous both spatially and spectrally. A transition is present for the 1.5-kHz center frequency (Figure 6C), where we can see an almost homogeneous BI decay for all distances. For stimuli with center frequency of 4 kHz (Figure 6D) the BI decay with the distance is almost independent of the bandwidth.

#### Direct-to-reverberant energy ratio

The DRR for PN and each noise band are plotted on Figures 6E–H as a function of the physical distance to the sound source. As it was exposed in Section Experiment 2: Apparent Source Distance for Broadband Noises, the room presents stronger reverberation for frequencies below 0.5 kHz and this contributes to lower the values of the DRR (the energy on the reverberant field becomes higher in proportion) for stimuli containing energy below this frequency. This is the main reason why, for a fixed bandwidth, as the center frequency becomes higher, the DRR values increases. Also, when the bandwidth increases the reverberant field gets more constant across distances yielding to a more homogeneous decay of the DRR.

#### Binaural room frequency response

**Figure 7:**
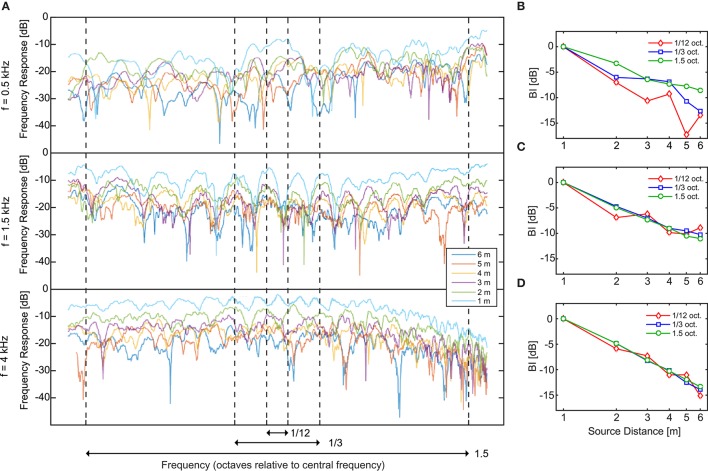
Analysis of the binaural room frequency response. **(A)** Binaural Room Frequency Response (BRFR) in dB, measured at the listener position for the six distances of the source (1–6 m), centered in the three stimulus frequencies (0.5, 1.5, and 4 kHz) in log scale. The vertical lines indicate the limits of the three stimulus bandwidths (1/12, 1/3, and 3/2 octave). The BRFR was obtained after Fourier transformation of the binaural impulse responses. **(B–D)** Integrating the BRFR in linear scale along the three different bands an independent measurement of the BI can be obtained. These BI values can be compared to the corresponding BIs obtained from the stimuli (Figures 6A–C). Approximated limits of the integration are shown as arrows at the bottom of **(A)**. The code symbols of different bandwidths are red diamonds, blue squares and green circles for 1/12, 1/3, and 1.5 octave, respectively.

In order to corroborate that the prominence of the resonances of the room for the lower frequencies is the main cause of the non-homogeneous decay of the BI, we calculated the frequency response of the room at the listener's ears: the binaural room frequency response (BRFR). In Figure 7A we display the BRFR of the room at the listener position for the six positions of the source, along the frequency range corresponding to the stimuli. The BRFR was obtained for each source position after Fourier transformation of the binaural room impulse response (BRIR) measured with the dummy head, as follows:

$$\begin{array}{lll} FR_{l,r}\left(f\right) & = & \frac{1}{L}\overset{L}{\underset{0}{\int }}h_{l,r}\left(t\right)e^{-i2\pi ft}dt\\ BRFR\left(f\right) & = & 10log_{10}\left[FR_{l}\left(f\right)FR_{r}\left(f\right)\right] \end{array}$$

where *h*_*l,r*_*(t)* correspond to the BRIR. This magnitude is computed in dB using an arbitrary reference and corresponds (up to a fixed constant in dB) to the BI elicited at the listener position for each frequency component in the room excited by the source for a given location. If a modal resonance is near that frequency component, it is expected that the BRFR will display large variations in its magnitude depending on the position, showing peaks if the source and listener are close to antinodes of the resulting standing wave, or valleys if they are close to nodal positions of the standing wave. This was the case of the low-frequency range, as can be appreciated in the two top panels of Figure 7A where the BRFR curves display strong variations in magnitude, with differences between peaks and valley as high as 30 dB. As the density of the modal resonances gets higher and the resonance peaks become shallower and overlap in the BRFR, the curve becomes smoother (see lower panel in Figure 7A).

From the curves displayed in Figure 7A it can also be seen that the narrower bandwidths were much more sensitive to the BRFR fluctuations. For the 1/12-oct. bandwidth and the two lower central frequencies, for example, a single normal mode of the room can alter the “normal” arrangement of the curves (from lower to higher distances). In this way, for such bands the BI dependence with distance can become non-monotonic. This can be tested by integrating the BRFR along each band. The resulting magnitude corresponds (up to a fixed constant in dB) to the BI for that band. These results are displayed in Figures 7B–D and can be compared to the corresponding BIs obtained from the recorded stimuli (Figures 6B–D). From this comparison, the non-monotonic BI curves (1/12 octave bandwidth for 0.5 and 1.5 kHz) obtained from the stimuli can be explained from the non-monotonic behavior of the integrated BRFR for the corresponding frequency bands.

### Correlations between acoustical cues and subjects' responses

To evaluate the relation between the previously obtained distance cues (BI and DRR) and the subjects' responses we calculated the partial correlation coefficients between the distance-dependent values of the cues and the mean distance responses (in logarithmic scale) of each subject. Partial correlation is the correlation between a given predictor variable and the dependent variable while holding contributions of all other predictor variables constant, and is required in this application because the predictor variables (BI and DRR) present a high degree of correlation with values ranging from 0.657 for the 0.5-kHz, 1/12-oct. band, to 0.997 for pink noise (multicollinearity).

**Figure 8:**
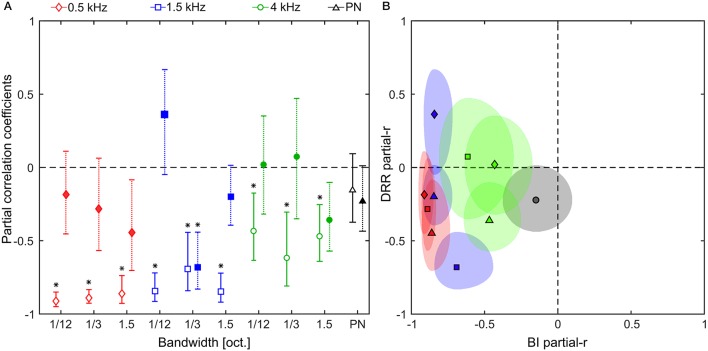
Partial correlation analysis. **(A)** Partial correlation coefficients between the log-scaled response and the: binaural intensity (open symbols); and DRR (filled symbols), for noise bands centered at 0.5, 1.5, and 4 kHz (red diamonds, blue squares, and green circles, respectively) and pink noise (black triangles). Error bars indicate confidence intervals at the 5% level. Asterisks indicate statistically significant (non-zero) correlations, analyzed by means of two-tailed one-sample *t*-tests. The overall level of significance was 5% adjusted by means of the Holm–Bonferroni correction. **(B)** Partial correlation coefficients showed in **(A)** represented as quasi-ellipses along x-axis (BI partial-r) and y-axis (DRR partial-r). The quasi-ellipses are centered on the mean partial correlation coefficients and the semi-axes indicate the confidence intervals at the 5% level. In **(B)** the center frequency is color-coded as in **(A)**, and the bandwidth is indicated as follows: diamond = 1/12 oct., square = 1/3 oct., triangle = 1.5 oct., and circle = PN.

In Figure 8A we show the across-subject average of the individual partial correlation coefficients between the subjects' log-responses and the binaural intensity controlling for the DRR; and between the log-responses and the DRR controlling for the binaural intensity, for each band and for PN. Two observations apply to all results. First, the BI showed a majority of negative correlation coefficients, which indicates that less intense noises are consistently associated with farther distances; and second, the DRR showed a more inhomogeneous pattern among noise bands, being positive in some cases, therefore not indicating a clear relation between the magnitudes.

In order to test whether the partial correlation coefficients were different from zero, we performed a set of two-tailed one-sample *t*-tests for each noise band (including pink noise) on the individual data. We found that the partial correlation is consistently lower than zero for BI (the only exception being PN) but not for DRR (the only exception being the 1.5-kHz, 1/3-oct. band), suggesting that, under the conditions of Experiment 2, the BI had a stronger and more reliable relation with the logarithm of the responses than the DRR.

A particularly striking case of this difference in the partial correlation coefficients occurs for the 0.5-kHz, 1/12-oct. band, where the correlation between BI and DRR is the lowest (*r* = 0.66, low collinearity). For this noise band the perceived auditory distance shows a non-monotonic increase with the distance. A similar non-monotonic behavior is observed for the binaural intensity as a function of distance, but not for the DRR curve. Moreover, the partial correlation between the log of the responses and the BI is high (r_BI_ = −0.91) while is low for DRR (r_DRR_ = −0.19), suggesting that the non-monotonic behavior of the responses can be explained by the non-monotonicity of the BI. For example, listeners could not perceive differences in distance for sources located at 3 and 2 m [mean reported distance 1.77 m, 95 % CIs (1.24, 2.30); and 1.94 m, 95 % CIs (1.41, 2.47), respectively, *p* = 0.63] despite the above-threshold (Larsen et al., 2008) change in DRR (BI = 0.71 and −3.11 dB, respectively). This suggests that the response could be explained by the fact that when the sound source gets farther, moving from 2 to 3 m, the BI remains almost equal (BI = −7.37 and −7.70 dB, respectively).

Another way of organizing the data is to plot the partial correlation for one cue against the other. This is shown in Figure 8B. This representation exposed the general pattern of association across stimuli. A two-tailed one-sample *t*-test showed that the average partial correlations between responses and BI are different than zero [*t*_(9)_ = −8.4, *p* = 1.5 × 10^−5^, Cohen's *d* = −2.65, 95% CIs (−3.95, −1.21)] while this is not the case for the DRR [*t*_(9)_ = −2.1, *p* = 0.07]. These results indicate that subjects tend to rely more consistently on the binaural intensity in order to judge the distance to a sound source.

## Discussion

The results obtained in this work indicate that the spectrum of a sound can significantly affect ADP of far-field (1–6 m) sound sources located in reverberant environments. Results of both psychophysical experiments showed an effect of the stimulus' frequency on the response for both pure tones and filtered noise bands: ADP was less accurate for stimuli containing energy mainly in the low-frequency range. In agreement with this, the three performance measures studied in Experiment 2 (correlation coefficient, slope, and standard deviation) were significantly affected by the center frequency of the auditory stimuli.

Unlike the clear effect of the center frequency, the effect of bandwidth was less straightforward. The results of Experiment 2 showed a non-significant main effect of bandwidth on the correlation coefficient. Similar results were obtained in the near field by Brungart (1999) and Kopčo and Shinn-Cunningham (2011). However, two complementary analysis suggested an effect of bandwidth on the response. First, for 1.5-kHz bands the correlation coefficient significantly increased with the bandwidth, as demonstrated by the positive slope obtained in the linear-regression analysis performed in Section Experiment 2: Apparent Source Distance for Broadband Noises. Second, the analysis of the slopes performed in Experiment 2 showed a significant effect of bandwidth for 0.5-kHz bands (the slope decreased as the bandwidth increased). These results indicate that, depending on the band center frequency, an increase in bandwidth induces different effects on the apparent distance of the source. The question arising from this observation is whether this effect is due to the change in the bandwidth *per se*, or to the inclusion and exclusion of certain frequency ranges in the signal as a consequence of changing the bandwidth. A rationale for the second hypothesis can be supported by the fact that increasing the bandwidth can enhance ADP performance, as seen in Figure 5A for 1.5-kHz bands, but can also worsen the response, as seen in Figure 5B for 0.5-kHz bands. The difference between the 0.5- and 1.5-kHz bands of the same bandwidth lies in the frequency region covered in each case. From the psychophysical data one could infer that the frequencies included when increasing bandwidth are beneficial for certain bands, while are detrimental for others.

Indeed, from the results displayed in Figure 7A it turns out that increasing the bandwidth has a different effect for 0.5-kHz bands compared to 1.5-kHz bands. Modal resonances are sparser at low frequencies and consequently the frequency response is more erratic in this range. This is most evident in the low-frequency region of the response for the 1.5-oct. band centered at 0.5 kHz, where the ordering of the frequency response curves with respect to source distance seems almost capricious (e.g., in some regions of the spectrum the frequency responses for sources located at *D* = 2 and 3 m is higher than for *D* = 1 m). Therefore, adding this frequency region, as the bandwidth increases, is certainly of little benefit for the reliability of binaural intensity as an accurate ADP cue. In fact, even when by increasing the bandwidth the BI decay with source distance becomes more monotonic, the bandwidth increase also entails a decrease in the slope of the integrated BI decay (Figure 7B) which can be linked to the effect of the bandwidth on the slope of the ADP response for 0.5-kHz bands (see Figure 5B). For the case of the 1.5-kHz bands, the possible explanation is less clear, since the ordering of the frequency-response curves with respect to source distance is also non-monotonic in certain regions. However, it is noteworthy that a substantial dip in the frequency response falls within the 1/12-octave bandwidth, hence compressing the response curves and reducing the possibility of discrimination between them. Therefore, for the 1.5-kHz bands, it is reasonable to expect an improvement in ADP as the bandwidth is increased. This is reflected in a clear increase in the linearity of the integrated BI decay (Figure 7C) while increasing the bandwidth from 1/12 to 1/3 oct. For 4-kHz bands, and since the frequency response shows a more homogeneous behavior, there is a much less significant increase of linearity of BI decay with bandwidth (Figure 7D).

Nevertheless, an increase in high-frequency content is not sufficient to obtain the ADP responses comparable to PN. As revealed by comparing the bands from Experiment 2 with PN, the performance for the stimuli with the higher frequency content (4-kHz bands) was lower than for PN, while the higher correlation coefficients were obtained for the 1.5-kHz, 1.5-oct. band and, certainly, PN. The common characteristic of these stimuli is that they contain energy both in the low (<1 kHz) and high (>2 kHz) regions of the audible spectrum (PN from 0.02 to 20 kHz, and the 1.5-kHz, 1.5-oct. band from 0.89 to 2.82 kHz). This result shows that, in order to obtain an accurate perception of the auditory distance in a room, not only high-frequency, but also low-frequency components are required. The former requirement allows the sound level to be less affected by the modal resonances of the room, and therefore provides a consistent (i.e., decreasing and monotonic) relation between target distance and sound intensity, while the later requirement contributes to the reverberant energy, reinforcing the DRR cue. This also implies that the minimum bandwidth required to obtain good ADP performance depends on the central frequency of the stimulus, since the 1.5-kHz, 1.5-oct. band showed a response similar to PN, while the 0.5- and 4-kHz bands of the same bandwidth did not.

In relation to the influence of the acoustical cues involved, the partial-correlation analysis suggests that, regardless of stimulus frequency and bandwidth, participants relied mostly on the BI rather than on DRR. This occurs even though the variation of the BI could lead to misjudgments of the source distance, and even though the variation of the DDR over the entire range of target distance was largely above threshold. This effect was evident in the response to 0.5-kHz bands where non-monotonic changes of the BI correlate well with the response. However, although BI appears to be a good candidate to explain the frequency-dependent effect of room resonant modes on ADP, the correlational approach of our analysis does not allow us to be conclusive about the exact contribution of DRR and BI in the obtained response. We consider then that future studies would be necessary where each of these cues could be manipulated in isolation to accurately study how BI and DRR are affected by the sound spectrum for far-field sources located in reverberant environments.

### Relation to past results

A direct comparison of our results with previous literature is not straightforward due to differences in methodology, stimuli characteristics, and acoustical cues involved. Although none of the studies that used several stimuli of different spectrum in both the near and the far field considered both the intensity and the DRR cues simultaneously, it is nonetheless interesting to look for coincidences and differences with our results.

Two rigorous studies where ADP was measured at different distances in response to various stimuli of different spectrum were conducted in the near field by Brungart (1999) and Kopčo and Shinn-Cunningham (2011). Although both studies show an effect of frequency on ADP, the reported effect was exactly the opposite to that obtained here: the correlation coefficient between distance and response was smaller for high-frequency stimuli than for the low-frequency ones (particularly for sources in front of the listener). In addition, both studies did not find a relationship between bandwidth and the response of listeners. These studies also show that the relative importance of the acoustical cues depends both on their availability and reliability. For example, Brungart (1999) found that amplitude-related cues dominate ADP in the median plane, while outside the median plane the distance perception depends primarily on low-frequency binaural cues. On the other hand, Kopčo and Shinn-Cunningham (2011) found that the response in a virtual semi-reverberant environment can be explained by assuming a simple relationship between the near-ear DRR and the mean distance judgments. It is difficult to compare the results of these studies with those obtained here mainly because both were made in the near field (where the low frequency ILD cue dominates) and the stimulus intensity was roved (with exception of the broadband stimulus in Brungart, 1999), excluding intensity from the available acoustical cues. In contrast, our results showed that, for the far field, stimuli containing only low-frequency components induced the lowest values of correlation coefficient.

As in all preceding studies, we found a systematic underestimation of the source distance for high-frequency stimuli (4-kHz bands, Figure 4C). There are two possible reasons to explain this underestimation. The first one is related with the decrease of high-frequency content, relative to low-frequency, when the sound travels through air. It is possible then that, like that reported by Coleman (1968), listeners have associated high-frequency stimuli with shorter distances to the source. The same hypothesis was elaborated by Butler et al. (1980). Although, in order to decrease this effect, we tested each type of stimulus in separate blocks, we cannot rule this hypothesis out. Another possible explanation is that the underestimation for 4-kHz bands was induced by the lower amount of reverberation (compared to wide-band noise) caused by the frequency response characteristic of the room (see Table 1). Previous studies have reported a systematic relationship between perceived distance and reverberation; therefore, the lower the reverberation, the closer the source is perceived. This explanation was also posed by Butler et al. (1980) to explain the greater effect of the frequency on the apparent distance obtained in an echoic, compared to an anechoic, environment. The results obtained for the 4 kHz bands are interesting, because they suggest that the spectrum can affect the ADP through the amount of reverberation present in the perceived stimulus. Here, the most accurate responses were obtained for stimuli containing energy both in the high and low regions of the audible spectrum, showing that reverberation was an important factor in the ADP response. However, as discussed before, reverberation *per se* does not guarantee an accurately-perceived distance.

Contrary to what was reported in previous studies (Butler et al., 1980; Nielsen, 1992), our results do not show an overestimation on the perceived distance for low-frequency stimuli. This discrepancy can be partially explained by methodological differences between our experiment and the previous ones. While in previous studies the amplitude of the stimuli was fixed at the ears of the listeners, in our experiment we let the intensity and the DRR vary (as it happens in a real environment) and, as we have shown, listeners' responses were mainly driven by intensity changes. For low-frequency stimuli, the reverberant energy is higher, hence the global intensity at the ears of the subject also increases. Therefore, even when more reverberation would induce an increase of the perceived distance, the rise of the global intensity dominates, inducing subjects to report shorter distances.

Our results showed that the resonant modes of the room strongly affected the apparent distance of the source for low frequency centered stimuli. Interestingly, for these stimuli, the room modes induced non-monotonic BI changes that correlate very well with the listeners' response. Previous works have shown that, in isolation, intensity provides more reliable distance information than DRR (Zahorik et al., 2005; Kolarik et al., 2013). However, in this case BI was not in isolation. In contrast, stimuli centered at 0.5 kHz induced the highest levels of reverberant energy within the room. Experiments by Kolarik et al. (2013) showed that for broadband sounds the perceptual weight of DRR as an ADP cue considerably increases in highly reverberant environments providing as accurate information as the intensity. Moreover, several studies have shown that ADP is most accurate when both DRR and intensity are available (Nielsen, 1992; Bronkhorst and Houtgast, 1999; Ronsse and Wang, 2012). While our results do not contradict those obtained in the aforementioned studies, they show that the relative influence of intensity and DRR cues in ADP also depends on the spectrum of the auditory stimulus. This fact is evident in the inaccurate responses obtained with noise-bands centered at 0.5 kHz despite that both the intensity and high levels of reverberation were available for the listeners. Perhaps the reason why DRR was not a reliable ADP cue in our study is that, for 0.5-kHz bands, the spectra of direct and reverberant sound closely resemble each other, making it difficult to discriminate between them. This could induce listeners to interpret reverberation as part of the direct sound. In connection to this possible explanation, there is also a debate about the ability of the nervous system to segregate the direct and reverberant sounds and compute the DRR. It was proposed that the auditory system derives this cue from the physical characteristics of the signal that covary with the direct and the reverberant sound, such as changes in the spectrum, temporal pattern, monaural changes in the spectral centroid or in frequency-to-frequency variability in the signal (Larsen et al., 2008) and interaural coherence (Bronkhorst, 2002). In this line, our results suggest that for reverberation to be an effective ADP cue, the auditory stimulus must contain energy in both the low and high regions of the spectrum.

Many previous studies support the idea that the presence of reverberation enhances the auditory perception of distance (Mershon and King, 1975; Mershon et al., 1989; Bronkhorst and Houtgast, 1999; Zahorik, 2002a,b; Kopčo and Shinn-Cunningham, 2011; Kolarik et al., 2015). However, the results obtained here show that, when the frequency and bandwidth of the stimuli are varied, it is not always true that more reverberation leads to a better estimation of the distance to a sound source. For example, we observed that reducing the frequency of the stimuli for a given bandwidth is always detrimental in terms of the accuracy of the response. This detrimental effect is, in part, a consequence of the existence of narrow, sparse and prominent resonant peaks in the frequency response of the room, that causes a non-monotonous behavior of the BI of the stimulus with distance. The magnitude of the effect and the frequency range will depend on the characteristics of the particular room, but for sufficiently low and narrow noise bands it will be very likely to find a negative effect of the reverberation on the accuracy of the distance estimate. Therefore, the benefit of DRR as an ADP cue cannot be generalized. This cue is useful also as long as the listener is able to discriminate between direct and reverberant sound, an issue that is not currently addressed in the literature. Further experiments are necessary to determine the influence of the spectrum on this ability and the effectiveness of the DRR cue for estimating auditory distance.

## Author contributions

IS, PE, and RV designed the study. IS, EC, EA, and RV performed the experiments. IS and ME performed the acoustical recordings and analysis. PE performed the statistical analysis of the behavioral data. IS, PE, ME, and RV wrote the paper.

### Conflict of interest statement

The authors declare that the research was conducted in the absence of any commercial or financial relationships that could be construed as a potential conflict of interest.
